# DNA methylation heterogeneity in complex tumor microenvironment: Quantitative methods, influencing factors, and clinical implications

**DOI:** 10.1016/j.gendis.2025.101832

**Published:** 2025-08-25

**Authors:** Yongle Xu, Shuangyue Ma, Manyi Xu, Hongbo Zhu, Yuncong Wang, Wenbo Dong, Jing Gan, Yusen Zhao, Xinrong Li, Shuangshuang Wang, Haoyu Hu, Jiaheng He, Shangwei Ning, Hui Zhi

**Affiliations:** a College of Bioinformatics Science and Technology, Harbin Medical University, Harbin, Heilongjiang 150081, China; b Liangzhu Laboratory, Zhejiang University, Hangzhou, Zhejiang 311121, China

**Keywords:** Circulating DNA, DNAmeH, Hemimethylation, Methylation biomarkers, Tumor microenvironment

## Abstract

5-Methylcytosine (5-mC) is the most prevalent DNA methylation modification in the human genome, and its abnormal patterns are strongly associated with tumor progression. Intratumoral and intertumoral DNA methylation heterogeneity (DNAmeH) primarily arises from cancer epigenome heterogeneity and the diverse cell compositions within the tumor microenvironment (TME). Furthermore, recent advancements in high-throughput sequencing and microarray technologies have facilitated the development of quantitative methods for measuring DNAmeH, enabling a more thorough exploration of the factors influencing it. Moreover, investigating various DNA methylation patterns at the single-cell level within the intricate TME sheds light on DNAmeH being driven by cellular heterogeneity. In addition, accumulating studies on the selection of methylation biomarkers in tissue or circulating DNA elucidate the cell specificity of DNA methylation, which is valuable for early cancer detection and personalized therapy. In this review, we elucidate the characteristics of intratumoral and intertumoral DNAmeH, considering DNAmeH differences across cancer types, among individual cells, and at allele-specific hemimethylation sites. Several metrics are summarized to quantitatively assess DNAmeH. We evaluate the factors that influence DNAmeH via these metrics, including the cell cycle phase, tumor mutational burden (TMB), cellular stemness, copy number variation (CNV), tumor subtype, tumor characteristics, tumor stage, state of tumor cells, hypoxia, and tumor purity. Finally, we highlight the deconvolution of TME cellular components and the application of predictive methylation biomarkers in cancer clinical research.

## Introduction

Tumors are complex diseases with high temporal and spatial heterogeneity characterized by tumor cells intermixed with normal cells in a unique proliferative environment known as the TME. The TME exhibits abnormal epigenetic changes more frequently than genetic mutations in the human genome.^1^^,^^2^ Recently, “nonmutational epigenetic reprogramming” has been identified as a novel hallmark of cancer.^3^ DNA methylation, a well-established epigenetic modification, demonstrates substantial plasticity in tumors. This plasticity, shaped by the enzymatic activities of DNA methyltransferases (DNMTs) and ten-eleven translocation (TET) dioxygenases, plays a critical role in tumor initiation and progression.^4^, ^5^, ^6^ Aberrant DNA methylation is often inherited by daughter cells, resulting in phenotypic differences characterized by hypermethylation and hypomethylation, which drive tumor evolution.^7^, ^8^, ^9^, ^10^, ^11^

DNA methylation has been shown to exhibit distinct cell- and tissue-specific patterns, with significant variability across tissues during tumorigenesis and embryonic development.^12^, ^13^, ^14^, ^15^, ^16^ In the TME, the DNA methylation pattern varies in distinct cell types and therefore shows intermediate methylation signals, reflecting DNAmeH attributed to cell-to-cell variations.^17^, ^18^, ^19^ DNAmeH is also observed in tumors due to the complexity of the TME and the tissue specificity of DNA methylation, which is correlated with different responses to and outcomes of tumor therapy among patients.^1^^,^^8^^,^^16^^,^^17^^,^^20^, ^21^, ^22^ Hence, understanding the nature of intra- and inter-tumor DNAmeH contributes to improving cancer treatments and monitoring tumor progression.^7^^,^^16^^,^^21^^,^^23^, ^24^, ^25^, ^26^

Advances in clinical research on tumor DNAmeH have centered on identifying DNA methylation biomarkers with tissue-specific methylation patterns. Specifically, circulating cell-free DNA (cfDNA) or tumor-derived circulating DNA (ctDNA), which are DNA fragments released by specific cells, is used to detect cell-specific Cytosine-phosphate-Guanine (CpG) sites, aiding in tumor diagnosis.^27^^,^^28^ Additionally, studies on factors contributing to DNA methylation alterations have revealed correlations between DNAmeH and genomic mutations, transcriptional heterogeneity, and tumor purity.^8^^,^^29^^,^^30^

Notably, quantitative measurements of DNAmeH can capture the methylation status of individual CpG sites, enabling exploration of tumor progression and prognosis.^8^^,^^21^^,^^31^ In this work, we outlined the following quantitative methods: (i) epiallele-based: epipolymorphism, methylation entropy (ME); (ii) discordant reads-based: the proportion of discordant reads (PDR), fraction of discordant read pairs (FDRP), and quantitative FDRP (qFDRP); (iii) methylation haplotype load (MHL); (iv) the proportion of sites with intermediate methylation (PIM); (v) DNA methylation inferred regulatory activity (MIRA). These metrics are applicable to quantitative DNA methylation analysis using technologies such as bisulfite sequencing or arrays. However, the dissection of DNAmeH at single-cell resolution is still limited by both in silico and experimental technology.^32^^,^^33^

Here, we first reviewed the characteristics of DNAmeH within the complex TME, considering its variation across diverse patient populations, various cancer types, and allele-specific levels. We then summarized the quantification methods for DNAmeH and examined the clinical implications of CpG biomarker identification. Furthermore, we investigated the associations between DNAmeH and both intrinsic and extrinsic factors. Our analysis of factors influencing DNAmeH offers novel insights, leveraging multi-omic data to elucidate cancer driver events without neglecting cellular heterogeneity.^8^^,^^16^^,^^17^^,^^22^^,^^34^ These findings contribute to the advancement of personalized therapeutic strategies for cancer patients.

## Cellular phenotypic plasticity of DNA methylation in tumors

Abnormal changes in DNA methylation are key factors in tumor initiation and metastasis, primarily manifesting as either aberrant hypomethylation or hypermethylation of regulatory regions in proto-oncogenes and tumor-suppressor genes.^35^, ^36^, ^37^, ^38^ These epigenetic changes orchestrate complex cellular phenotypic plasticity, including chromatin remodeling, metabolic rewiring, and immune escape. One key mechanism is the silencing of tumor-suppressor genes via promoter hypermethylation. For instance, hypermethylation of the TP53 promoter suppresses its transcriptional activity, thereby accelerating the progression of hepatocellular carcinoma (HCC).^39^ Similarly, in endometrial carcinoma, MLH1 promoter methylation induces mismatch repair deficiency (dMMR), promoting microsatellite instability and oncogenesis.^40^ In glioblastoma and head and neck squamous cell carcinoma, MGMT hypermethylation impairs DNA repair mechanisms and contributes to chemoresistance.^41^ Conversely, hypomethylation events frequently activate proto-oncogenes. The dysregulation of the proto-oncogene MYC in aggressive tumor cells is linked to hypomethylation at CTCF-binding sites within enhancers.^42^ Likewise, in lung adenocarcinoma, PRAME promoter hypomethylation at TEAD1-binding sites enhances epithelial–mesenchymal transition (EMT) and metastasis.^43^ Global hypomethylation predominantly targets repetitive DNA elements, driving distinct oncogenic processes across cancer types. In colorectal cancer (CRC), hypomethylation of the long interspersed nuclear element-1 (LINE-1) repetitive sequence is associated with chromosomal instability and metastatic behavior,^44^ while alpha-satellite DNA (SATα) hypomethylation in prostate cancer promotes chromatin de-condensation and oncogene activation.^45^ These observations reflect a broader epigenetic mechanism whereby DNA methylation orchestrates chromatin architecture. Specifically, global hypomethylation of repetitive elements such as LINE-1 and Alu induces chromatin decondensation, thereby facilitating aberrant transcription factor binding and oncogene activation.^46^ In contrast, CpG island hypermethylation recruits methyl-binding proteins and histone deacetylases (e.g., MeCP2, HDACs), leading to chromatin compaction and silencing of tumor-suppressor genes.^46^, ^47^, ^48^, ^49^

Aberrant DNA methylation also influences tumor immunity. For instance, the demethylation of promoters in effector genes affects the activation of cytotoxic T lymphocytes,^50^^,^^51^ while DNMT3a regulates the fate of CD8^+^ T cells, and its knockdown favors differentiation towards memory precursor cells instead of effector T cells.^52^ Furthermore, global methylation changes in cancer-associated fibroblasts (CAFs) affect gene expression within the transforming growth factor-beta (TGF-β) pathway, modulating their pro-tumor activities.^53^ In summary, DNA methylation is critical for regulating cancer-related genes and reprogramming cellular phenotypes.^1^^,^^53^^,^^54^

## DNAmeH

### Intra-tumor DNAmeH

In the human genome, DNA methylation predominantly occurs at the fifth carbon of the cytosine pyrimidine ring, forming 5-methylcytosine (5-mC).^55^ Given its central role in regulating gene expression and cellular identity, precise mapping of 5-mC is critical. Accordingly, current DNA methylation detection technologies can be categorized into two major strategies based on the detection scope: genome-wide detection and locus-specific detection. Alternatively, based on the detection technologies employed, they can be divided into three major categories: bisulfite conversion, microarray-based hybridization, and enrichment-based techniques. Detailed technical parameters and application scenarios are summarized in Table 1.

**Table 1:** DNA methylation detection methods.

Method	Detection Scope	Detection Principle	Typical Applications	Reference
Infinium BeadChips (27K/450K/EPIC 850K)	Locus-specific	Microarray-based hybridization	Epigenome-wide association study (EWAS), large-scale screening	Bibikova et al,^58^ Bibikova et al,^59^ Pidsley et al^60^
RRBS	Locus-specific	Bisulfite conversion	Targeted profiling of promoters and CpG islands	Nakabayashi et al^61^
Targeted-BS	Locus-specific	Bisulfite conversion	Validation of candidate loci	Reinders et al^62^
oxBS-seq	Depends on coupled method	Bisulfite conversion	5hmC functional studies	Booth et al^63^
TAB-seq	Depends on coupled method	Bisulfite conversion	Base-resolution mapping of 5hmC	Yu et al^64^
WGBS	Genome-wide	Bisulfite conversion	Comprehensive methylome profiling	Cokus et al^65^
MeDIP-seq	Genome-wide	Enrichment-based	Global methylation profiling, cfDNA methylation analysis	Weber et al^66^
MBD-seq	Genome-wide	Enrichment-based	Regional methylation quantification	Serre et al^67^
MRE-seq	Genome-wide	Enrichment-based	Complementary analysis with MeDIP/MBD	Maunakea et al^68^

Infinium BeadChips (27K/450K/EPIC 850K): Infinium HumanMethylation27 BeadChip, Infinium HumanMethylation450 BeadChip, and Infinium MethylationEPIC BeadChip, RRBS: Reduced Representation Bisulfite Sequencing, Targeted-BS: Targeted Bisulfite Sequencing, oxBS-seq: Oxidative Bisulfite Sequencing, TAB-seq: Tet-Assisted Bisulfite Sequencing, WGBS: Whole-Genome Bisulfite Sequencing, MeDIP-seq: Methylated DNA Immunoprecipitation Sequencing, MBD-seq: Methyl-CpG Binding Domain Sequencing, MRE-seq: Methylation-sensitive Restriction Enzyme Sequencing.

**Figure 1:**
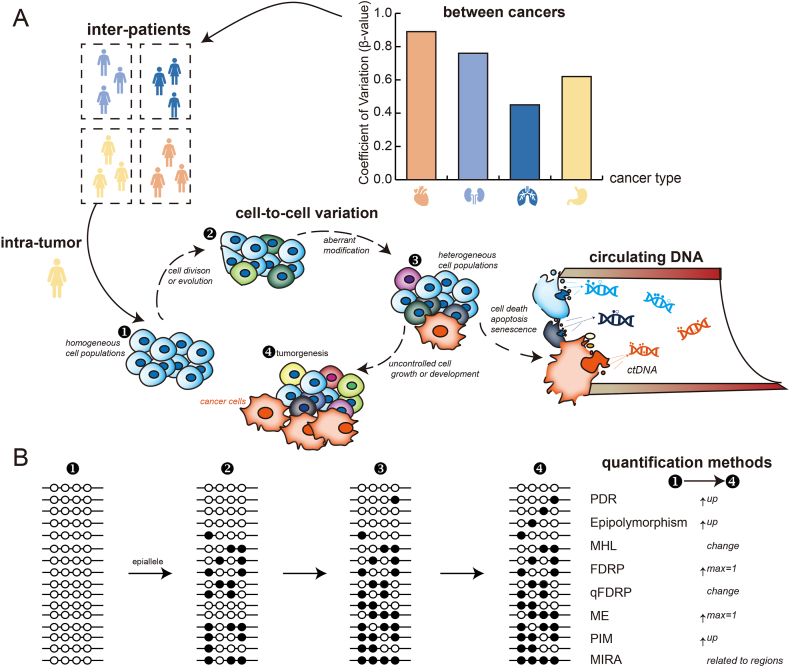
Mechanisms underlying the formation of DNAmeH and its quantification methods. **(A)** This schematic illustrates the progression of DNAmeH from inter-patient and intra-tumor heterogeneity, detailing stages of cell division, aberrant methylation, cellular heterogeneity, and tumorigenesis. The release of ctDNA is shown as a consequence of cell apoptosis, necrosis, and senescence. **(B)** The quantification methods for DNAmeH include PDR, epipolymorphism, MHL, FDRP, qFDRP, ME, PIM, and MIRA. Solid circles represent methylated CpG sites, and empty circles indicate unmethylated CpG sites.

These technological advances have revealed the fundamental characteristics of DNA methylation patterns. At the single-cell level, the methylation status of individual CpG sites is binary, being either methylated (1, representing 5-mC) or unmethylated (0, representing C). However, most DNA methylation profiles exhibit a bimodal distribution (near 0 and 1), with a notable number of CpG sites found in an intermediate state, particularly in bulk tissue samples.^17^^,^^56^ This phenomenon occurs primarily because the methylation value of a given CpG site represents an averaged signal across heterogeneous cell populations, reflecting cellular heterogeneity within the complex TME (Fig. 1A).^17^^,^^56^^,^^57^ Consequently, higher intermediate methylation levels indicate stronger intra-tumor DNAmeH, facilitating the analysis of cell compositions in the TME and enabling the identification of specific CpG sites as biomarkers.

### Inter-tumor DNAmeH

DNA methylation is tissue-specific, demonstrating differential methylation levels in diverse tumors (Fig. 1A).^12^, ^13^, ^14^, ^15^, ^16^^,^^69^ Sheffield et al assessed differences in DNA methylation levels across cancers using the coefficient of variation (CV) of β-values, which reflects the epigenetic heterogeneity among cancer types.^17^ De et al quantified DNA methylation differences between patients using the interquantile range (IQR).^56^ Both CV and IQR capture the variability in methylation values,^70^ indicating that higher CV or IQR values signify greater methylation differences among samples. Additionally, studies have revealed dynamic DNA methylation regulation across cancers. Yang et al identified three CpG sites among 15 cancer types, where β-values showed different correlations with the expression of BVES and PRDM1, genes involved in tumor cell growth.^71^, ^72^, ^73^ Liu et al discovered tumor cluster-specific hypomethylated CpG sites across 12 cancers. In adenocarcinoma, these CpG sites were linked to the TP53 signaling pathway, while in squamous carcinoma, they were associated with the TGF-β and Wnt signaling pathways.^74^

### Hemimethylation

Hemimethylation is a novel epigenetic marker, referring to asymmetrically methylated CpG sites, where the parent strand is methylated and the complementary nascent strand remains unmethylated during DNA replication.^75^, ^76^, ^77^, ^78^ Chao et al reported that hemimethylated CpG sites in the repetitive sequence satellite 2 DNA (Sat2) of ovarian epithelial tumors were consistently present on the same DNA strand orientation, hinting at their potential use as metrics for monitoring tumor progression.^79^ Sun et al demonstrated that continuously hemimethylated CpG sites exhibited distinct methylation patterns in non-small cell lung cancer (NSCLC) patients compared to those in normal samples and that these hemimethylated sites were associated with genes involved in regulating tumor cell growth.^76^ They further identified hemimethylated CpG sites that not only impact tumor cell growth but also serve as transition markers for monitoring tumor progression.^80^ Additionally, hemimethylation is cell-specific and is stably inherited due to interactions of DNMTs during cell division.^81^ Notably, DNMT1 preferentially targets hemimethylated CpG sites to mediate the methylation of nascent cytosines, while DNMT3a and DNMT3b are responsible for *de novo* methylation, where 5-methyl groups are added to symmetrically unmethylated cytosines (Fig. 1).^78^^,^^81^, ^82^, ^83^, ^84^ Advances in next-generation sequencing have enabled separate measurement of DNA methylation levels on forward and reverse strands, facilitating hemimethylation research.^80^ Hua et al developed sequencing methods named ssg-MeDIP-Seq and sscf-MeDIP-Seq, specifically for measuring semi-methylation in genomic DNA and cell-free DNA (cfDNA), respectively. They demonstrated that semi-methylation serves as an independent biological marker and can be used in conjunction with differentially methylated regions to build models, achieving better performance.^85^

### Quantifications of DNAmeH

Most studies measure the average DNA methylation signal at specific CpG sites across complex cell populations, often neglecting cell-to-cell variations.^86^ Methylation detection techniques, such as WGBS and RRBS, are capable of capturing DNA methylation at single-CpG resolution.^8^^,^^86^ Table 2 presents a selection of quantitative methods for assessing DNAmeH (Fig. 1B).

**Table 2:** Overview of DNAmeH quantification methods.

Method	Formula	Reads	Value	R package	Reference
PDR	$\frac{num\left(dr\right)}{num\left(ar\right)}$		1/3	WSH	Landau et al^8^
Epipolymorphism	$1-\sum_{i=1}^{16}p\left(x_{i}\right)$		2/3	Methclone	Landan et al^34^
ME	$\frac{-1}{4}\sum_{i=1}^{16}p\left(x_{i}\right)\log_{2}\;p\left(x_{i}\right)$		1/3	Methclone	Xie et al^87^
FDRP	$\frac{num\left({rp}_{differ}\right)}{num\left(rp\right)}$		0.396	WSH	Schermer et al^86^
qFDRP	$\frac{\sum_{i=1}^{rp}\frac{num\left(M_{differ}\right)}{4}}{num\left(rp\right)}$		2/3	WSH	Schermer et al^86^
MHL	$\frac{\sum_{i=1}^{4}{mh}_{Cm}}{\sum_{l=1}^{3}l+1},l=0$		0.4528	WSH	Guo et al^88^
PIM	$\frac{num\left(M_{inter}\right)}{num\left(M\right)}$	–	–	Epihet	Sheffield et al^17^
MIRA	$\log\frac{\left(flank\right)}{\left(mid\right)}$	–	–	MIRA	Sheffield et al^17^

*dr*: discordant reads, *ar*: all reads, rp: reads-pairs, ${rp}_{differ}$: reads-pairs with inconsistent methylation, $M_{differ}$: different methylated CpG sites in rp, Cm: methylated CpG site, ${mh}_{Cm}$: methylated CpG site in methylation haplotype, $M_{inter}$: CpG sites with intermediate methylation state, *flank*: DNA methylation level of the flank region, *mid*: DNA methylation level of the center region.

### Proportion of discordant reads (PDRs)

The proportion of discordant reads (methylated cytosine and unmethylated cytosine in the same read) at consecutive CpG sites is defined as the PDR value.^8^^,^^89^ A larger PDR indicates more discordant reads covering the locus, suggesting chaotic intercellular methylation patterns.^8^^,^^89^ PDR values fluctuate in accordance with tumor cell division, indicating an association between DNAmeH and genomic instability and tumor progression.^7^, ^8^, ^9^, ^10^ The dynamic alterations in DNA methylation patterns within heterogeneous cell populations contribute to tumor evolution and are associated with poor patient survival outcomes.^7^, ^8^, ^9^, ^10^^,^^21^^,^^90^

### Epipolymorphism

Epipolymorphism is determined based on epialleles (four adjacent CpG sites and 16 kinds of epialleles are shown in Fig. 1B), suggesting that the cell populations are composed of various epialleles.^34^ An increase in the number of epiallele categories leads to a higher degree of epipolymorphism, signifying greater diversity in DNA methylation patterns.^34^^,^^86^^,^^91^ The maximum epipolymorphism value is 0.9375 when all 16 epialleles are present.^22^ The probability of epialleles can be calculated via methclone.^34^^,^^86^^,^^91^

### Methylation haplotype load (MHL)

Methylation haplotype load (MHL) is derived from the DNA methylation haplotype (MH), which is defined as groups of adjacent CpG sites with consistent methylation status.^26^^,^^88^^,^^92^ The MHL score quantifies the proportion of fully methylated CpG sites across all combinations of read-substrings in each MH.^86^ The maximum MHL value is 1, indicating that all reads are fully methylated across the MH. Conversely, the minimum MHL value is 0, indicating that all reads are fully unmethylated.^86^

### Fraction of discordant read pairs (FDRPs)

Fraction of discordant read pairs (FDRPs) represent the percentage of paired reads with discordant methylation patterns at the same CpG sites. The maximum FDRP value is 1, indicating that all paired reads have discordant methylation patterns, while the minimum FDRP value is 0, indicating that all paired reads have identical methylation patterns.^86^

### Quantitative FDRP (qFDRP)

In contrast to FDRP, quantitative FDRP (qFDRP) quantifies discordance by utilizing the Hamming distance, which counts the number of CpG sites with differing methylation statuses on paired reads (Fig. 1; Table 1).^86^ The calculations of both FDRP and qFDRP require binary alignment (BAM) files generated from raw sequencing reads.^86^^,^^93^ The maximum qFDRP value is 1, indicating that the methylation patterns of all paired reads are discordant, while the minimum qFDRP value is 0, indicating that all paired reads have identical methylation statuses.^86^

### Methylation entropy (ME)

Methylation entropy (ME) is calculated based on the frequency distribution of different epialleles via Shannon entropy.^86^^,^^87^ The minimum ME value is 0, indicating consistent methylation patterns across all epialleles at a specific locus. The maximum ME value is 1, indicating a high diversity of epiallele categories at this locus (Fig. 1).^86^^,^^87^

### Proportion of sites with intermediate methylation (PIM)

The DNA methylation level of individual CpG sites in a single cell type displays a bimodal distribution, while the intermediate methylation level is elevated in heterogeneous cell populations.^17^^,^^56^ The proportion of sites with intermediate methylation (PIM) serves as a metric of cellular heterogeneity within tissues.^16^^,^^17^^,^^56^ Higher PIM scores indicate increased levels of DNAmeH.^17^^,^^56^ Additionally, intermediate methylation CpG sites are identified through two methods: (i) applying a Bayesian bimodal distribution to detect CpG sites with β-values within the 95% confidence interval^17^; (ii) categorizing methylation levels to calculate the proportion of CpG sites exhibiting intermediate methylation.^16^

### DNA methylation inferred regulatory activity (MIRA)

Methylation-based inference of regulatory activity (MIRA) is defined to quantify the regulatory activity of specific regions by aggregating genome-wide DNA methylation data with common biological annotations into a new methylation profile.^17^^,^^94^ Higher MIRA scores indicate stronger regulatory activities at specific genomic regions, such as transcription factors (TFs), CpG islands (CGIs), and enhancers, which typically exhibit lower levels of DNA methylation.^17^^,^^21^^,^^90^

### Influential drivers of DNAmeH

DNAmeH exhibits considerable variation in different cancer types, and this heterogeneity is influenced by a multitude of both intrinsic and extrinsic factors. Building on insights from previous studies, we used the PIM metric to investigate intra- and inter-tumor DNAmeH in 33 cancer types using data from the TCGA and CGGA databases. Several key factors have been shown to be closely associated with changes in DNAmeH.

## Intrinsic factors

### DNAmeH in relation to cell cycle regulation

**Figure 2:**
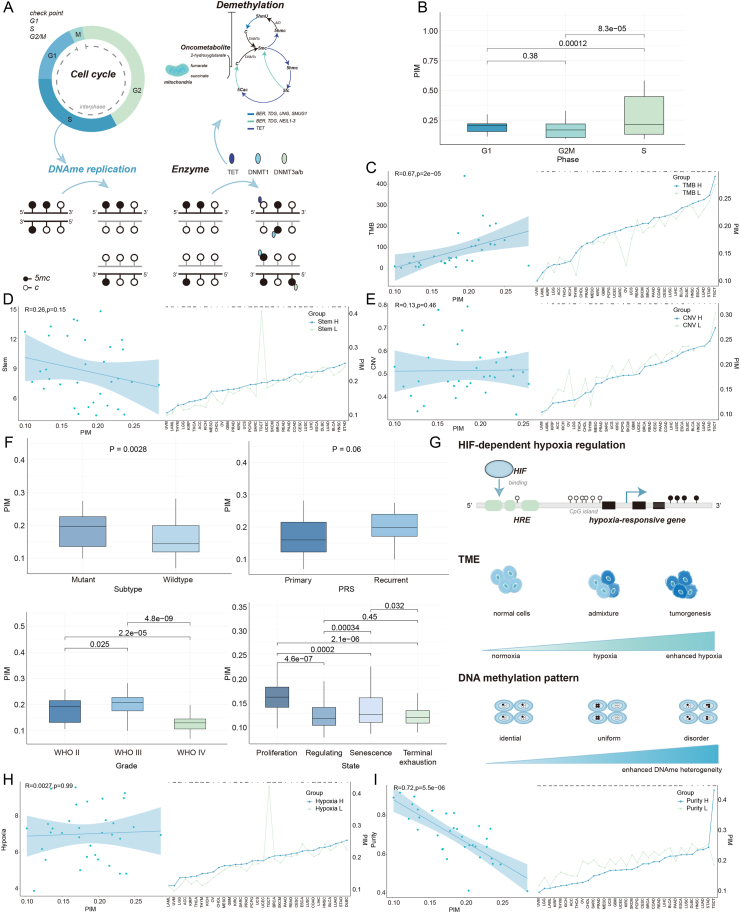
Influential Drivers of DNAmeH. **(A)** Schematic representation of the cell cycle and DNA methylation–demethylation dynamics, showing the roles of various enzymes, including TET, DNMT1, DNMT3A, and DNMT3B, in modulating DNA methylation during DNA replication. **(B)** Boxplots showing DNAmeH levels across distinct cell cycle phases (G1, G2/M, S). **(C**–**E)** Scatter plots showing the correlations of DNAmeH with TMB, stemness, and CNV across 33 cancer types, with shaded regression lines indicating trend significance. Additional line graphs demonstrate the relationships between DNAmeH and TMB, stemness, and CNV metrics within specific cancer types. **(F)** Boxplots showing PIM comparisons across distinct genetic subtypes, tumor grades (WHO classifications II, III, IV), recurrence status, and cellular functional states (proliferation, regulation, senescence, terminal exhaustion), with *p*-values indicating statistical significance of differences (the GBM bulk RNA-seq and methylation array datasets used for the analysis were downloaded from the CGGA data portal). **(G)** Illustration of the biological process by which hypoxia regulates DNAmeH. **(H, I)** Scatter plots showing the associations between DNAmeH and hypoxia levels or tumor purity, with shaded regression lines indicating trend significance. Additional line graphs demonstrate the relationships between DNAmeH and hypoxia or purity metrics within specific cancer types.

Dynamic changes in DNA methylation are influenced by the regulation of cell cycle checkpoints (CCPs) and the activity of enzymes.^4^^,^^5^^,^^30^ CCP regulation is vital for DNA damage repair and monitoring replication errors, and deregulation of CCPs is associated with the formation and progression of various cancers.^95^^,^^96^ In particular, the DNMT and TET families mediate the addition and removal of 5-methyl groups (Fig. 2A).^4^^,^^5^^,^^75^ During DNA replication, the 5-methyl groups present on the original DNA strand are copied to the newly synthesized daughter strands.^75^ Johnson et al suggested that deregulation of CCPs may lead to an accumulation of DNA mutations, further driving increased DNA methylation disorder.^30^ Brown et al indicated that global DNA methylation levels differ between the G1 and S phases of the cell cycle.^97^ To provide further evidence, we characterized DNAmeH across different cell cycle phases in gliomas via PIM. Consistent with previous studies,^97^, ^98^, ^99^ during the S phase, the methylation maintenance mechanisms in cancer cells are frequently disrupted during DNA replication, leading to aberrant methylation patterns in newly synthesized DNA strands. This disruption contributes to heightened DNAmeH, which is closely associated with tumor initiation and progression. The G1 phase, a critical stage for the regulation of gene expression, is marked by the hypermethylation of tumor suppressor genes such as CDKN2A and TP53, resulting in their functional loss and promoting tumor progression. In contrast, by the G2/M phase, DNA replication has been completed, and methylation patterns tend to stabilize (Fig. 2B).

### CNV as a driver of epigenetic instability

CNV is a pivotal marker of genomic instability, with an increased CNV burden potentially disrupting DNA methylation patterns and thereby exacerbating epigenetic heterogeneity within tumors.^100^ Although we did not observe a significant correlation between CNV and inter-tumor DNAmeH across 33 cancer types, 73% (24/33) of cancer types demonstrated a significant increase in intra-tumor DNAmeH in regions with high CNV burden (Fig. 2C). This association is further substantiated by the studies of Johnson et al.^30^^,^^101^ These findings hint that an elevated CNV burden may amplify DNAmeH by increasing genomic instability.

### TMB and its impact on DNAmeH

TMB serves as a key indicator of the cumulative mutational load within tumor genomes. It is intimately linked to genomic instability, progressive mutation accumulation, and the generation of neoantigens.^102^ Research underscores that TMB affects tumorigenesis at the genomic level and reshapes the immune microenvironment by modulating epigenetic mechanisms, particularly through DNAmeH.^103^ Luo et al elucidated a significant correlation between TMB and specific CpG methylation configurations, suggesting that TMB bolsters the effectiveness of immunotherapy by altering methylation patterns.^103^ Furthermore, in gliomas, a high TMB is correlated with poorer prognosis and impacts immune responses through modifications in specific DNA methylation patterns.^104^ In our assessment of inter-tumor and intra-tumor DNAmeH across 33 cancer types, a significant correlation was observed between inter-tumor DNAmeH and TMB (*R* = 0.67, *P* = 2e-05). Additionally, in 73% (24/33) of the cancer types, high-TMB samples showed higher intra-tumor DNAmeH than low-TMB samples (Fig. 2D). This finding is consistent with prior studies, suggesting that mutation accumulation may mediate epigenetic reconfiguration via mechanisms such as DNA methylation.^105^

### Stemness characteristics and DNA methylation stability

Stemness characteristics represent a crucial factor influencing tumor progression in tumor cells. Numerous studies have demonstrated a strong association between stemness and DNAmeH in tumor cells.^106^ Although the relationship between stemness characteristics and inter-tumor DNAmeH across 33 cancer types (*R* = −0.26, *P* = 0.15) was disturbed by multiple factors, our findings revealed that in 79% (26/33) of the cancer types, samples with high stemness exhibited lower intra-tumor DNAmeH levels compared to those with low stemness (Fig. 2E).^107^ This trend implies that stem cells, while maintaining their undifferentiated state, exhibit a relatively stable epigenetic profile, whereas differentiation increases DNAmeH, likely in response to the requirements of new functional roles and epigenetic regulation.

### Cancer subtypes, recurrence, staging, and cellular states: correlation with intra-tumor DNAmeH

Studies have demonstrated that distinct methylation patterns may be regulated by factors such as tumor subtypes, recurrence, staging, and cellular biological states.^30^^,^^108^ We further investigated the relationships between these factors and intra-tumor DNAmeH in the glioma landscape.^109^ Our findings revealed that DNAmeH in gliomas with isocitrate dehydrogenase (IDH) mutations was significantly higher than that in IDH-wildtype gliomas (*P* = 0.0028) (Fig. 2F), consistent with studies by Johnson et al.^30^ Additionally, DNAmeH was generally higher in recurrent glioma samples than in primary samples (*P* = 0.06) (Fig. 2F), reflecting accumulated genetic and epigenetic changes during tumor progression and indicating increased diversity in methylation patterns throughout recurrence.^108^ Among glioblastoma multiforme (GBM) samples at different stages, DNAmeH also exhibited significant differences: stage III samples had the highest DNAmeH, followed by stage II, with stage IV samples showing the lowest DNAmeH (Fig. 2F). This trend may reflect changes in heterogeneity and methylation patterns across stages of tumor progression.

Using the TCellSI tool,^110^ we categorized GBM samples into four cellular states (“Proliferation”, “Regulating”, “Senescence”, and “Terminal-exhaustion”) (Fig. 2F). The results showed that samples in the proliferation state had the highest DNAmeH, followed by those in the senescence state, while those in the regulating and terminal-exhaustion states exhibited lower DNAmeH. Previous studies have suggested that cells in the proliferation state, characterized by frequent DNA replication and division, may experience increased genomic perturbation, thereby elevating DNAmeH.^111^ The higher DNAmeH observed in the senescence state reflected genomic instability, disrupted epigenetic regulation, and diversified gene expression patterns associated with cellular aging.^112^ In contrast, cells in the regulating state, with specific functional demands and stable gene expression, exhibit lower DNAmeH.^113^ Terminal-exhaustion cells showed the lowest DNAmeH level due to functional exhaustion, markedly reduced metabolic activity, and a more stabilized or “stagnant” state of gene expression and epigenetic regulation.^114^ In summary, tumor recurrence, staging, subtype, and the biological state of tumor cells are all closely related to DNAmeH.

## Extrinsic factors

### Role of hypoxia in modulating DNAmeH

Hypoxia is a biological feature of a large number of tumors and is correlated with tumor progression, the TME, and decreased activity of the demethylation enzyme TET.^18^^,^^115^, ^116^, ^117^ The expression of hypoxia-responsive genes is primarily regulated by the hypoxia-inducible factor (HIF) and is associated with DNA methylation of the HIF binding element (Fig. 2G).^116^^,^^118^ A comprehensive evaluation across 33 cancer types revealed that tumors with higher levels of hypoxia are often accompanied by increased inter-tumor DNAmeH. This trend is particularly pronounced in intra-tumor comparisons, where in 85 % (28/33) of cancer types, samples with high hypoxia showed elevated DNAmeH compared to those with low hypoxia (Fig. 2H). Hypoxic conditions induce a marked reduction in TET enzyme activity, thereby impairing DNA demethylation. Consequently, there is an elevation in DNA hypermethylation, leading to an increase in DNAmeH both within individual tumors (intra-tumor) and among different tumors (inter-tumor).^30^^,^^119^, ^120^, ^121^

### Influence of tumor purity on DNAmeH

Distinct cellular constituents within the TME display markedly heterogeneous DNA methylation profiles. The proportion of malignant cells within the TME, termed tumor purity, plays a pivotal role in the precision and interpretation of DNA methylation data.^16^ Zheng et al developed an algorithm that leverages high-throughput DNA methylation data to infer tumor purity, which demonstrated that DNA methylation profiles can effectively estimate tumor purity across various cancer samples.^122^ This revelation underscores the significance of tumor purity in the context of methylation data analysis and hints at a potential correlation between tumor purity and DNAmeH. Through both inter-tumor and intra-tumor analyses encompassing 33 cancer types, we observed a significant negative correlation between tumor purity and inter-tumor DNAmeH (*R* = −0.73, *P* = 5.5e–06). Meanwhile, 94% (31/33) of the cancer types showed lower intra-tumor DNAmeH in samples with high tumor purity than in those with low purity (Fig. 2I). These findings suggest that the presence of non-tumor cells may disrupt the DNA methylation landscape of tumor cells, ultimately leading to an elevation in the DNAmeH of tumors.

## Advances in DNAmeH related to tumor research

### Methylation-based deconvolution assesses TME compositions

Accumulating evidence suggests that cell type mixing has an impact on the measurement of DNA methylation.^123^, ^124^, ^125^ DNA methylation variability can be primarily attributed to the cellular proportions inferred from methylation profiles.^125^, ^126^, ^127^ A closely related topic is the EWAS, which aims to correct cellular composition effects in heterogeneous samples, such as blood or tissue, to explore correlations between DNA methylation and disease phenotypes.^126^, ^127^, ^128^, ^129^ Methylation-based deconvolution approaches have been proposed for assessing cell composition in the complex TME, including (i) Epigenetic Dissection of Intra-Sample Heterogeneity (EpiDISH), which evaluates cell proportions within samples by identifying cell-specific CpG sites^130^; and (ii) MethylCIBERSORT, a CIBERSORT-based method for genome-wide DNA methylation data, which analyzes TME complexity by estimating cellular components and tumor purity.^131^ These methods facilitate the comparison of inter-sample differences without ignoring cellular heterogeneity bias.

### DNA methylation markers in ctDNA for clinical applications

CfDNA is fragmented DNA released during cell apoptosis, necrosis, or senescence; it is termed ctDNA when specifically released from tumor cells.^12^^,^^55^^,^^132^, ^133^, ^134^ An increasing number of studies have explored specific methylation signatures of cfDNA to dissect the TME and investigate the clinical variables of tumor patients. For example, (i) Guo et al identified tissue-specific CpG haplotypes with stable methylation patterns to infer tumor cell compositions^88^; (ii) tissue-specific CpG sites served as cancer early detection biomarkers, which predicted more accurately than Alpha-Fetoprotein (AFP, a marker for HCC) and carcinoembryonic antigen (CEA, a marker for CRC) for tumor stages^23^^,^^26^; (iii) in mPCa patients undergoing drug therapy, most variable probes (MVPs) showed significant DNA methylation changes^25^; (iv) in CRC patients with liver metastasis receiving neoadjuvant chemotherapy, cfDNA methylation levels dynamically varied between responders and non-responders, serving as early indicators of sensitivity to chemotherapy.^135^ In summary, cell- and tissue-specific cfDNA methylation markers hold potential for early cancer diagnosis and treatment response prediction.

### Identification of cell- and tissue-specific biomarkers for cancer treatment

DNAmeH has been revealed to be associated with tumor development and clinical factors. Specifically, (i) the aggressiveness of prostate cancer (PCa) and esophageal squamous cell carcinoma (ESCC) is associated with increased DNAmeH^21^^,^^90^^,^^136^^,^^137^; (ii) abnormal DNA methylation status of intestinal epithelial cells leads to morphological changes, thereby stimulating the development and progression of colon cancer^138^; and (iii) quantitative scores PDR and MIRA are linked to clinical variables in acute myeloid leukemia, chronic lymphocytic leukemia, and Ewing sarcoma.^8^^,^^17^^,^^31^ Moreover, DNA methylation exhibits considerable intra- and inter-tumor heterogeneity due to the high diversity of methylation patterns in somatic cells, which accelerates cancer cell evolution.^7^, ^8^, ^9^, ^10^ Consequently, numerous studies have identified cell- and tissue-specific methylation biomarkers for cancer treatment. For example: (i) in NSCLC patients undergoing anti-PD-1 therapy, the methylation levels of candidate CpG sites were higher in non-responders than in responders, predicting response outcomes more accurately than the expression of programmed death-ligand 1 (PD-L1)^139^; (ii) in metastatic melanoma patients receiving anti-PD-1 therapy, specific CpG sites served as potential biomarkers, displaying hypomethylation in patients with slower tumor progression^140^; (iii) in breast cancer (BRCA) patients treated with bevacizumab (a targeted molecular drug), CpG biomarkers associated with progression-free survival demonstrated differential methylation levels between responders and non-responders, distinguishing patients with better therapeutic outcomes^141^; and (iv) in BRCA patients, the methylation levels of CpG sites related to immune pathways significantly changed after radiotherapy.^142^ A selection of studies identifying cell- and tissue-specific CpG sites for cancer prognosis is summarized in Table 3.

**Table 3:** Cell- and tissue-specific DNA methylation markers for cancer clinical analysis.

Methylation	CpG sites	Cancer	Pattern	Tissue	Gene	Reference
Tissue-specific	cg17213048	*HCC*	*Hyper*	HCC ctDNA	ATAD2	Xu et al^26^
Tissue-specific	cg10673833	*CRC*	*Hyper*	CRC tissues	MYO1G	Luo et al^23^
Tissue-specific	cg07610777	*HCC*	*Hyper*	HCC cfDNA	GJB6	Hlady et al^143^
Tissue-specific	cg16051361	*Pca*	*Hyper*	Pca cfDNA	RAB21	Gordevičius et al^144^
Tissue-specific	cg03313364	*Pca*	*Hyper*	Pca cfDNA	STAT5A	Gordevičius et al^144^
Cell-specific	cg12673499	*CRC*	*Hyper*	CD8^+^ T cell	TRIM8	Zou et al^145^
Cell-specific	cg24088496	*BRCA*	*Hyper*	NK cell	MAML2	Chen et al^146^
Cell-specific	cg17124583	*BRCA*	*Hyper*	B Cell	GATA3	Chen et al^146^
Cell-specific	cg08708961	*BRCA*	*Hyper*	Neutrophil	PSEN2	Chen et al^146^

HCC: hepatocellular carcinoma, CRC: colorectal cancer, BRCA: breast cancer, Pca: prostate cancer, NK: natural killer cell, Hyper: hypermethylation.

## Discussion

DNA methylation, a crucial epigenetic modification, is integral to tumorigenesis and progression.^147^ Abnormal DNA methylation patterns, driven by the activity of DNMTs, are inherited by daughter cells during cell division and contribute to the clonal expansion of cancer cells. Both intra-tumor and inter-tumor DNAmeH have emerged as a focal point in understanding cancer biology and improving clinical outcomes.

Intra-tumor DNAmeH, specifically, refers to the variation in DNA methylation patterns across different cells within a single tumor. This phenomenon indicates the cellular heterogeneity within the TME, which is a hallmark of cancer evolution. Specific CpG sites exhibit differential methylation statuses within tumor cells,^16^^,^^17^^,^^56^ offering insights into the complex interactions among cancer cells, stromal cells, and the immune microenvironment. DNAmeH contributes to tumor progression by driving clonal evolution and influencing the response to therapy.^147^ Recent advancements in DNA methylation-based deconvolution methods, such as EpiDISH and MethylCIBERSORT, have improved assessments of cellular composition and tumor purity by analyzing the methylation profiles of distinct cell types.^130^^,^^131^ These techniques allow a more precise dissection of tumor heterogeneity and facilitate the identification of specific methylation biomarkers for guiding treatment strategies.^12^^,^^88^^,^^130^^,^^131^ Inter-tumor DNAmeH, on the other hand, emphasizes the variability in methylation patterns across distinct tumors. This variability can impact factors such as the tumor mutational burden, immune microenvironment composition, and drug resistance—elements critical to treatment efficacy and disease progression.^135^^,^^139^, ^140^, ^141^

Quantitative metrics of DNAmeH, such as PDR, PIM, and MIRA, are crucial for examining tumor epigenetic heterogeneity and its correlations with clinical variables, including tumor subtype, stage, and treatment response.^8^^,^^17^^,^^21^^,^^31^^,^^90^ These metrics not only reveal tumor heterogeneity but also demonstrate correlations with patient outcomes, thereby underscoring the potential of DNA methylation as a powerful prognostic and predictive biomarker.

DNAmeH is shaped by a complex interplay between intrinsic and extrinsic factors, each significantly influencing methylation patterns and their effects on tumor phenotypes. Intrinsic factors, including cell cycle regulation, CNV, and TMB, shape DNAmeH by promoting genomic instability and mutation accumulation.^4^^,^^30^^,^^97^^,^^100^^,^^102^ Furthermore, extrinsic factors such as hypoxia and tumor purity influence DNAmeH by modulating enzymatic activity (e.g., inhibition of TET enzymes under hypoxic conditions) and altering the cellular composition of the TME.^18^^,^^115^

This complex and dynamic nature of DNAmeH holds substantial clinical potential. Studies indicate that elevated DNAmeH levels within tumors correlate with disease progression and recurrence risk, particularly in cancers such as breast cancer and PCa, where DNAmeH serves as a predictor of patient survival and therapeutic outcomes.^90^^,^^139^^,^^141^ Inter-tumor heterogeneity further impacts personalized treatment approaches, with methylation states in cfDNA and ctDNA functioning as non-invasive biomarkers for early detection and real-time monitoring of tumor dynamics, thereby facilitating the development of individualized treatment strategies.^23^^,^^26^

Despite significant advancements in DNAmeH research,^16^^,^^17^^,^^90^^,^^148^, ^149^, ^150^ several challenges remain in integrating DNAmeH research into clinical practice. One major obstacle is the difficulty of measuring DNAmeH at the single-cell level, as single-cell bisulfite sequencing is constrained by limited read depth and coverage, particularly in complex tumor samples.^150^, ^151^, ^152^, ^153^ Furthermore, although deconvolution methods have significantly advanced cellular composition analysis in tumors, there is a pressing need for more robust algorithms and standardized protocols to improve precision and reproducibility in DNAmeH analysis.^32^^,^^33^

Future research should prioritize improving the accuracy of DNAmeH quantification, particularly at the single-cell resolution. Increasing technical replicates or developing targeted sequencing strategies for specific methylation hotspots could refine our understanding of DNAmeH’s role in tumor evolution. Moreover, integrating DNA methylation data with genomic, transcriptomic, and proteomic profiles could provide a more comprehensive understanding of tumor biology and treatment responses.^7^^,^^147^ The potential of DNAmeH to drive personalized cancer therapy is immense. With a deeper understanding of how DNA methylation patterns influence tumor behavior and treatment outcomes, epigenetic biomarkers could be harnessed to optimize drug selection, adjust treatment regimens, and predict patient responses more effectively.

## CRediT authorship contribution statement

**Yongle Xu:** Writing – original draft, Writing – review & editing, Visualization, Supervision, Resources, Investigation, Data curation, Validation, Software, Methodology, Formal analysis. **Shuangyue Ma:** Writing – original draft, Validation, Software, Visualization, Supervision, Data curation. **Manyi Xu:** Writing – review & editing, Visualization, Supervision, Data curation, Writing – original draft, Validation, Software. **Hongbo Zhu:** Writing – review & editing, Data curation. **Yuncong Wang:** Writing – original draft, Data curation. **Wenbo Dong:** Writing – original draft, Data curation. **Jing Gan:** Writing – original draft, Data curation. **Yusen Zhao:** Writing – original draft, Data curation. **Xinrong Li:** Writing – original draft, Data curation. **Shuangshuang Wang:** Writing – original draft. **Haoyu Hu:** Writing – original draft. **Jiaheng He:** Writing – original draft. **Shangwei Ning:** Supervision, Conceptualization, Writing – review & editing, Project administration. **Hui Zhi:** Writing – review & editing, Supervision, Methodology, Funding acquisition, Conceptualization, Project administration, Investigation, Formal analysis.

## Data availability

Thirty-three cancer bulk RNA-seq datasets and methylation array datasets were downloaded from the Cancer Genome Atlas Program data portal (TCGA, https://www.cancer.gov/tcga). Additionally, the GBM bulk RNA-seq and methylation array datasets were downloaded from the Chinese Glioma Genome Atlas database portal (CGGA, https://www.cgga.org.cn/index.jsp).

## Funding

This work was supported by the 10.13039/501100001809National Natural Science Foundation of China (No. 32170674).

## Conflict of interests

The authors declare no conflict of interests.
